# Long-term imaging reveals behavioral plasticity during *C. elegans* dauer exit

**DOI:** 10.1186/s12915-022-01471-4

**Published:** 2022-12-13

**Authors:** Friedrich Preusser, Anika Neuschulz, Jan Philipp Junker, Nikolaus Rajewsky, Stephan Preibisch

**Affiliations:** 1 Berlin Institute for Medical Systems Biology (BIMSB), Max Delbrück Center for Molecular Medicine in the Helmholtz Association (MDC), 10115 Berlin, Germany; 2 Institute for Biology, Humboldt University of Berlin, 10099 Berlin, Germany; 3 Janelia Research Campus, Howard Hughes Medical Institute, Ashburn, VA 20147 USA

**Keywords:** Behavioral imaging, *C. elegans dauer*, Neuroplasticity

## Abstract

**Background:**

During their lifetime, animals must adapt their behavior to survive in changing environments. This ability requires the nervous system to undergo adjustments at distinct temporal scales, from short-term dynamic changes in expression of neurotransmitters and receptors to longer-term growth, spatial and connectivity reorganization, while integrating external stimuli. The nematode *Caenorhabditis elegans* provides a model of nervous system plasticity, in particular its dauer exit decision. Under unfavorable conditions, larvae will enter the non-feeding and non-reproductive stress-resistant dauer stage and adapt their behavior to cope with the harsh new environment, with active reversal under improved conditions leading to resumption of reproductive development. However, how different environmental stimuli regulate the exit decision mechanism and thereby drive the larva’s behavioral change is unknown. To fill this gap and provide insights on behavioral changes over extended periods of time, we developed a new open hardware method for long-term imaging (12h) of *C. elegans* larvae.

**Results:**

Our WormObserver platform comprises open hardware and software components for video acquisition, automated processing of large image data (> 80k images/experiment) and data analysis. We identified dauer-specific behavioral motifs and characterized the behavioral trajectory of dauer exit in different environments and genetic backgrounds to identify key decision points and stimuli promoting dauer exit. Combining long-term behavioral imaging with transcriptomics data, we find that bacterial ingestion triggers a change in neuropeptide gene expression to establish post-dauer behavior.

**Conclusions:**

Taken together, we show how a developing nervous system can robustly integrate environmental changes activate a developmental switch and adapt the organism’s behavior to a new environment. WormObserver is generally applicable to other research questions within and beyond the *C. elegans* field, having a modular and customizable character and allowing assessment of behavioral plasticity over longer periods.

**Supplementary Information:**

The online version contains supplementary material available at 10.1186/s12915-022-01471-4.

## Background

Animals adapt their behavior in response to changes in the environment, a phenomenon also referred to as behavioral plasticity [1]. However, timescales of behavioral adaptation can vary drastically. While some stimuli elicit a fast and short response (e.g., escape), others will influence behavior over minutes (e.g., foraging) or several hours (e.g., day and night cycles). Therefore, characterizing the effect of the environment on an organism's behavior requires the integration of multiple temporal scales, comprising both short responses and long-term adaptation processes.

Studying the adaptation of neuronal circuits and the resulting behavioral dynamics requires experimental tractability, i.e., the possibility to identify, trace, and compare neuronal circuits across genetic backgrounds and conditions. While this is challenging in complex mammalian circuits, the nematode *Caenorhabditis elegans (C. elegans)* has emerged as a suitable model organism for studying the relationship between nervous system plasticity, sensory inputs, and behavior by possessing a stereotyped and fully mapped [2], yet flexible [3] nervous system.

**Fig. 1:**
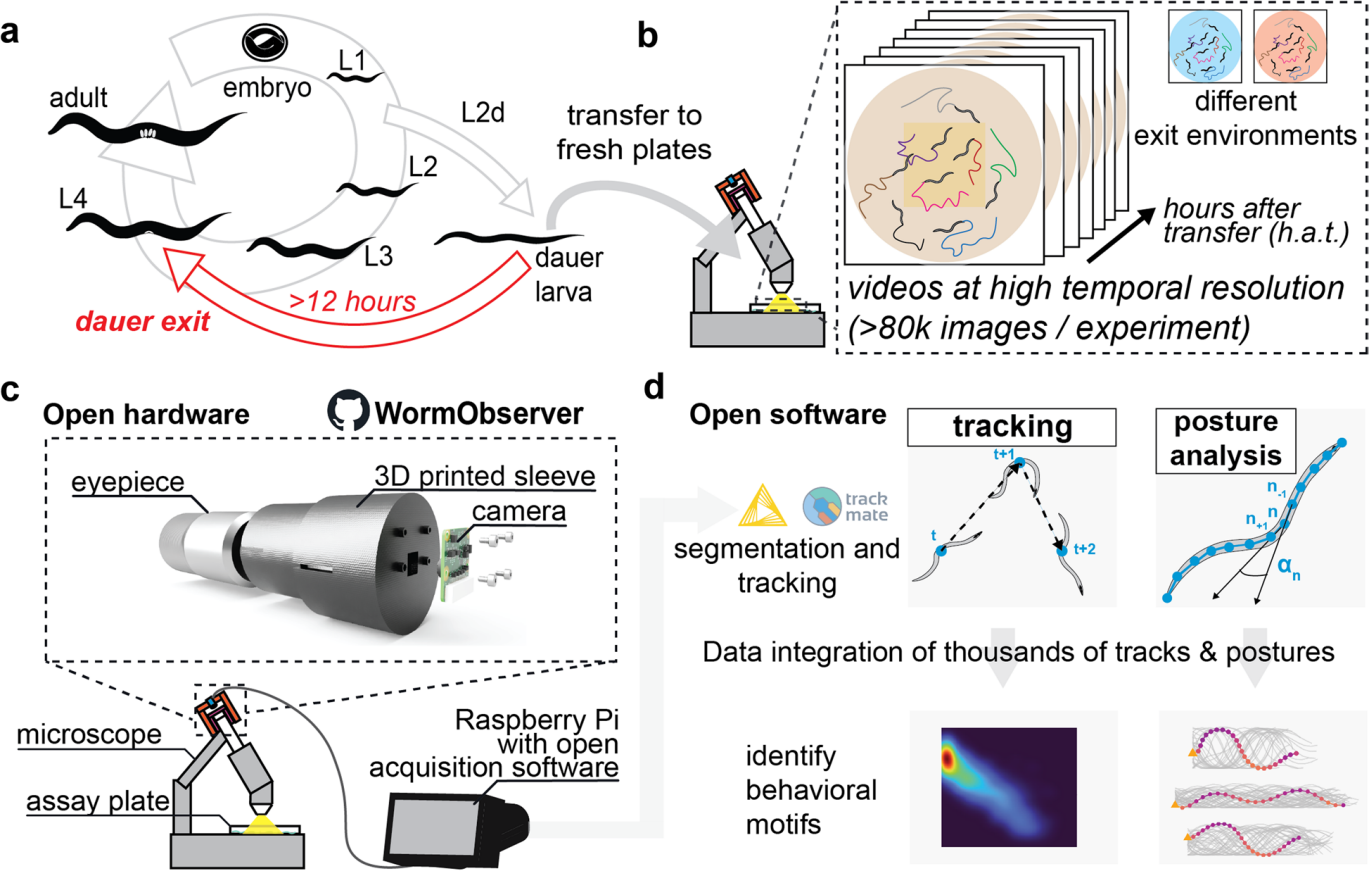
Overview. **a** Dauer exit is a developmental decision to resume reproductive development, which takes more than 12 h, highlighting the need for long-term behavioral imaging. **b** Overview of the assay used in this study. Dauer larvae are transferred to a new environment (i.e., a fresh plate) and their change in behavior is recorded across several hours. Time resolved measurements will be relative to the time point when worms were transferred to the new environment (hours after transfer or *h.a.t.*). **c** Sketch of the open hardware; a 3D printed solution for acquiring timelapse videos through the eyepiece of a stereoscope. Camera and acquisition parameters are controlled by a Raspberry Pi, which streams the data to a workstation for data processing. **d** Summary of the software, which integrates KNIME and the TrackMate framework. By integrating data from thousands of tracks and worm postures, users can build robust, quantitative models of *C. elegans* behavior

The *C. elegans* dauer diapause is a *bona fide* example of a developmental switch that requires neuronal plasticity (Fig. 1a). When encountering an unfavorable environment, e.g., that is lacking a food source, *C. elegans* L1 larvae can actively deviate from the canonical reproductive life cycle and develop into the so-called dauer larva [4]. This alternative developmental stage is particularly stress resistant and geared for survival, notably by possessing a thickened, closed cuticle. Dauer larvae cease the uptake of food and can remain in this dormant state for several months [5]. Importantly, once environmental conditions improve, dauer larvae will sense and integrate that changed environment and resume reproductive development.

Previously, receptors, signaling molecules, and transcription factors involved in dauer formation have been studied extensively (for review see [6] and [7]). At the neuronal level, individual remodeling events specific to the dauer larva have been identified [8–12]. However, a quantitative and temporally resolved description of the behavioral adaptation process is missing but needed to contextualize molecular and structural changes. Ultimately, linking behavioral adaptation to specific molecular programs (e.g., changes in gene expression) will improve our understanding how a developing nervous system is able to remain plastic and integrate external stimuli.

Here, we introduce a new open-hardware and open-source platform for quantitative behavioral analysis of *C. elegans.* Although solutions for imaging *C. elegans* behavior have been previously developed [13–17], performing long-term imaging of hundreds of larvae over several hours to extract population dynamics remains challenging. Our WormObserver platform combines long-term population imaging of *C. elegans* larvae and open source analysis software in a modular, end-to-end solution that can be adapted to other research questions (Fig. 1b–d). We use the platform to describe the behavioral trajectory of dauer exit and characterize dauer-specific behavioral motifs. Moreover, we identify environmental signals that initiate the exit decision and correlate the resulting changes in behavior with gene expression dynamics.

## Results

### WormObserver: an open hardware solution for long-term behavioral recordings

To capture behavioral changes during the dauer exit decision, a process previously shown to take more than 12 hours [4], we developed the WormObserver, a lightweight, low-cost, open hardware imaging solution that allows parallel tracking of hundreds of worms on culture plates. The WormObserver platform is based on a standard stereoscope, making use of high-quality optical components already available in most laboratories. We designed and 3D printed a two-component adapter that fits the eyepiece of a stereoscope (Fig. 1c, Methods) and holds an attached camera. The camera is connected to a Raspberry Pi, a commercially available, credit-card sized computer, which enables automated video acquisition [18]. To provide users with a ready-to-use solution, we developed a graphical user interface (GUI) for easily adapting imaging parameters like acquisition frame rate, resolution, and timing of the videos to be acquired. To capture behavioral motifs at high temporal resolution over more than 12 h, we split the timelapse in short video sequences (8 min), thereby facilitating tracking and analysis. Acquired videos are streamed directly to a workstation PC applying a custom, open source image processing workflow that uses KNIME [19], ImgLib2 [20], and the TrackMate framework [21] (Methods). Taken together, combining a 3D printed adapter and off-the shelf hardware components with a standard stereoscope and custom software, we envision our tool to be broadly applicable for a large group of users, not only to specialists in microscopy or image analysis. The WormObserver platform is documented [22] and image acquisition and image feature extraction workflows can be executed through graphical user interfaces.

### Characterizing behavioral adaptation during dauer exit

Dauer larvae have been described to be distinct in their behavioral repertoire and previous studies have improved our understanding of the underlying neuronal plasticity [9, 11, 23, 24]. Dauers exhibit increased bouts of prolonged locomotor quiescence but can be highly mobile and are able to nictate [25], a dauer-specific behavior that increases the chances to hitch-hike on other animals to escape an unfavorable environment in natural contexts [26, 27]. Moreover, dauers moving on a plate generally appear less bent and stiffer compared to well-fed larvae of the same age [28]. Nevertheless, a time-resolved characterization of dauer-exit specific changes in behavior is missing but needed to answer three key questions: First, it is unclear for how long the worm is integrating the changed environment before adapting its behavior. Second, which behavioral motifs are changing during this adaptation process and are all behavioral adaptations occurring at once or in a stepwise manner? And third, are the different environmental stimuli known to regulate dauer exit (i.e., food stimulus, pheromone) all affecting the same or different aspects of behavior?

To answer these questions, we focused on the first 12 h of dauer exit for characterizing behavioral changes and used the condition lacking a food source as a negative control in which dauers cannot successfully exit. We reasoned that behavioral adaptation related to the exit decision would occur before the onset of growth, previously described to resume after ca. 14 h of dauer exit [4]. We induced the dauer stage by a combination of standardized starvation, crowding, and high-temperature conditions (see Methods). Next, extracted dauer larvae were shifted from starved to fresh plates with or without a bacterial food source and monitored for 12 h. To compare behavior across conditions, we refer to each time point as hours after transfer *(h.a.t.)* to a fresh plate (Fig. 1b).

### In a new environment, dauers will switch from behavioral quiescence to a highly mobile state

**Fig. 2:**
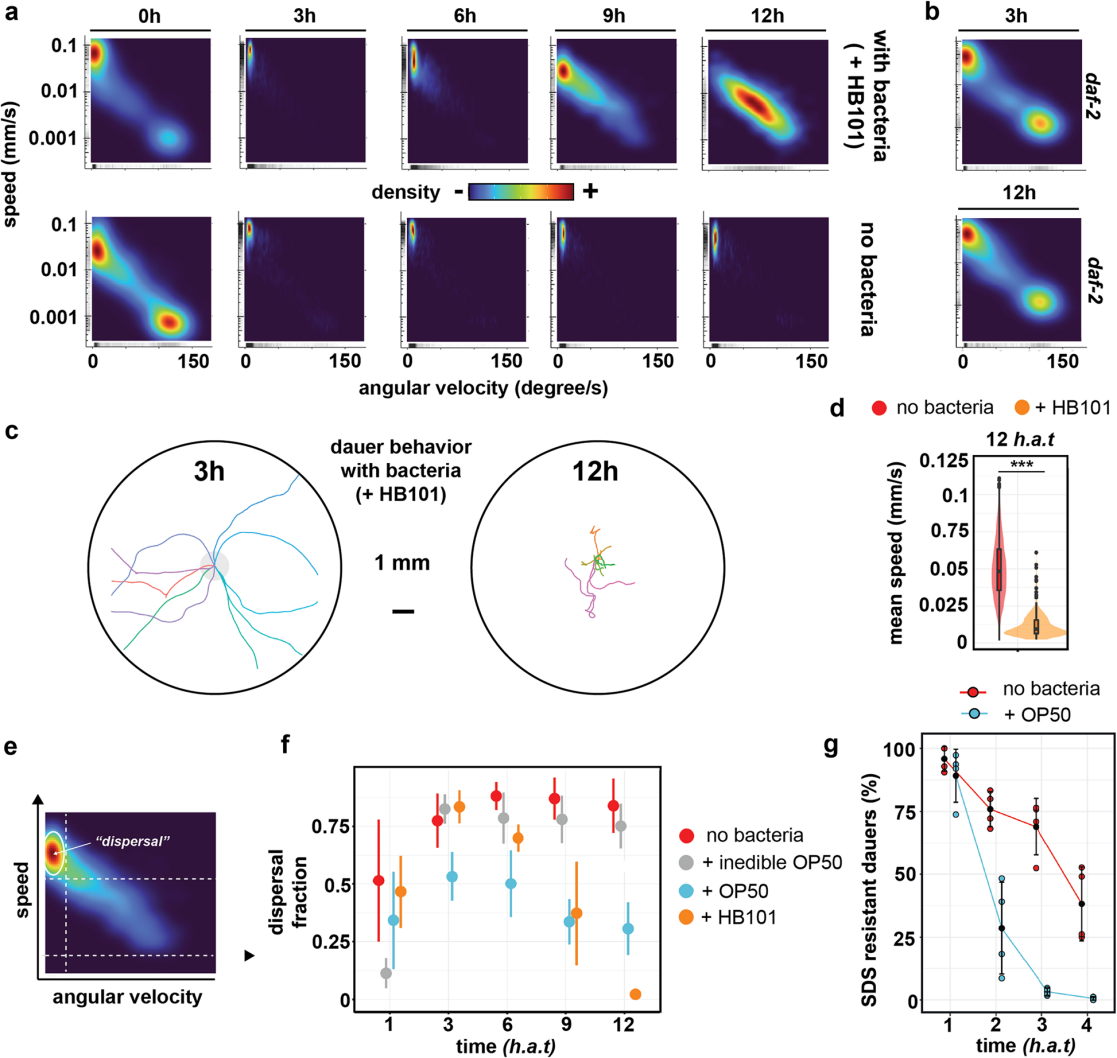
A food-dependent behavioral switch during dauer exit. **a** Two-dimensional probability distributions of speed (mm/s) and angular velocity (degrees/s) averaged over 10 s time windows across the first 12 h of dauer exit, highlighting the effect of a food source on behavior. Data is from *n* = 3 experiments and each time point contains data from at least 250 tracks (average number of tracks = 1262). **b** Two-dimensional probability distributions obtained from *daf-2* dauers on +OP50 NGM plates, *n* = 2 experiments. **c** Dauer behavior before and after the food-dependent behavioral switch. Shown are 10 randomly selected example tracks of a length between 2 and 4 min. **d** Mean speed (calculated per track) at *12 h.a.t.*. *** indicates *p*-value < 0.0001 (Mann-Whitney test). **e** Schematic summarizing how the dispersal phenotype was defined, based on thresholds for speed and angular velocity (see Methods). **f** Relative abundance of dispersal behavior as defined in **e**. Error bars depict standard deviation of at least 2 experiments. **g** Percentage of dauers that survive SDS treatment at different time points during dauer exit. This assay can be used as a proxy for inferring onset of food ingestion. Dots represent experimental replicates

We first asked the question whether the speed by which the worms move on the plate was changing during the course of dauer exit. As expected, dauers that were transferred to a new environment initially exhibited very slow movement or no movement at all, characterized by high angular speed (i.e., path curvature) and low speed. The previously described dauer specific locomotion quiescence [23] was observed for dauers in all tested conditions and is independent of the presence of bacteria on the plate (Fig. 2a, Additional file 1: Supplemental Figure S1; Supplemental Figure S2). However, many larvae remained highly mobile (high speed) and moved straight in one direction (low angular velocity). This initial division into a quiescent and a highly mobile regime appeared in all our experiments and is also recapitulated when quantifying behavior of *daf-2* dauer larvae that cannot exit the dauer stage (Fig. 2b, Additional file 1: Supplemental Figure S1). We therefore conclude that dauer larvae, when put onto fresh plates, remain quiescent but can explore the environment in a specific dispersal mode characterized by fast movement into one direction. This is also consistent with the observation that dauers can be highly mobile [28]. Moreover, when extending our analysis beyond the first hours after transfer, we noticed that the quiescent fraction disappeared 3 *h.a.t*. At this time point, all animals displayed a strong dispersal phenotype characterized by high speed and low angular velocity (Fig. 2c, d). This was similar across the tested conditions, although the exact timing varied, with the notable exception of *daf-2* dauer larvae, where a significant fraction remained in the quiescent phenotype (Fig. 2b, Additional file 1: Supplemental Figure S1). Taken together, we observe that independent of a bacterial food source, 3 h after being exposed to a new environment, wild type dauer larvae exhibit a first behavioral switch from quiescence to a highly mobile dispersal-like behavior. Importantly, this first behavioral switch is independent of the presence of a food source and was observed in all tested conditions.

### A second behavioral switch depends on a food stimulus

When extending our analysis beyond the first three hours, we noticed a second behavioral adaptation that was dependent on the presence of an edible food source. After being exposed to the new environment for approximately 6 h, larvae that were placed on a high quality food source decreased speed but increased turning frequency, resembling canonical behavior of adult worms [29] (Fig. 2a). After 12 h, dauer specific dispersal behavior was completely abolished (Fig. 2c, d). In contrast, dauers placed on plates lacking food continued moving at high speed and low angular velocity and did not exit dispersal behavior (Fig. 2a, d). When quantifying the relative fraction of dispersal behavior across conditions and time points (Fig. 2e), we noticed that the behavioral switch from dispersal to dwelling started to occur with high quality (HB101) and low quality food (OP50), in both cases after 6 *h.a.t*. (Fig. 2f). Given this independence of the behavioral adaptation process on food source quality, we performed all remaining experiments on OP50. Interestingly, larvae placed on bacteria that were previously treated with an antibiotic, making them too large to be ingested by the worm, did not exit the dauer-specific dispersal behavior (Fig. 2f). This finding suggested that the ingestion of bacteria is crucial for inducing the behavioral switch. However, when developing into the dauer stage, larvae cease pharyngeal pumping and develop a buccal plug that prevents them from uptake of external components (e.g., a bacterial food source) [4]. To further investigate this, we confirmed that the ability to ingest external components is restored prior to 6 *h.a.t.* during dauer exit, however with delayed timing if a food source is lacking (Fig. 2g). Taken together, we have characterized a second behavioral switch that consists in exiting the dauer-specific dispersal phenotype and that is, in contrast to the first switch, dependent on the presence of a bacterial food source and therefore indicative of successful dauer exit. Furthermore, we note that it is the ingestion (and not the presence) of bacteria that drives this behavioral change, matching the observation that by the time the switch occurs, dauers will have resumed food ingestion.

### Posture adaptation is independent of the presence of a food source

**Fig. 3:**
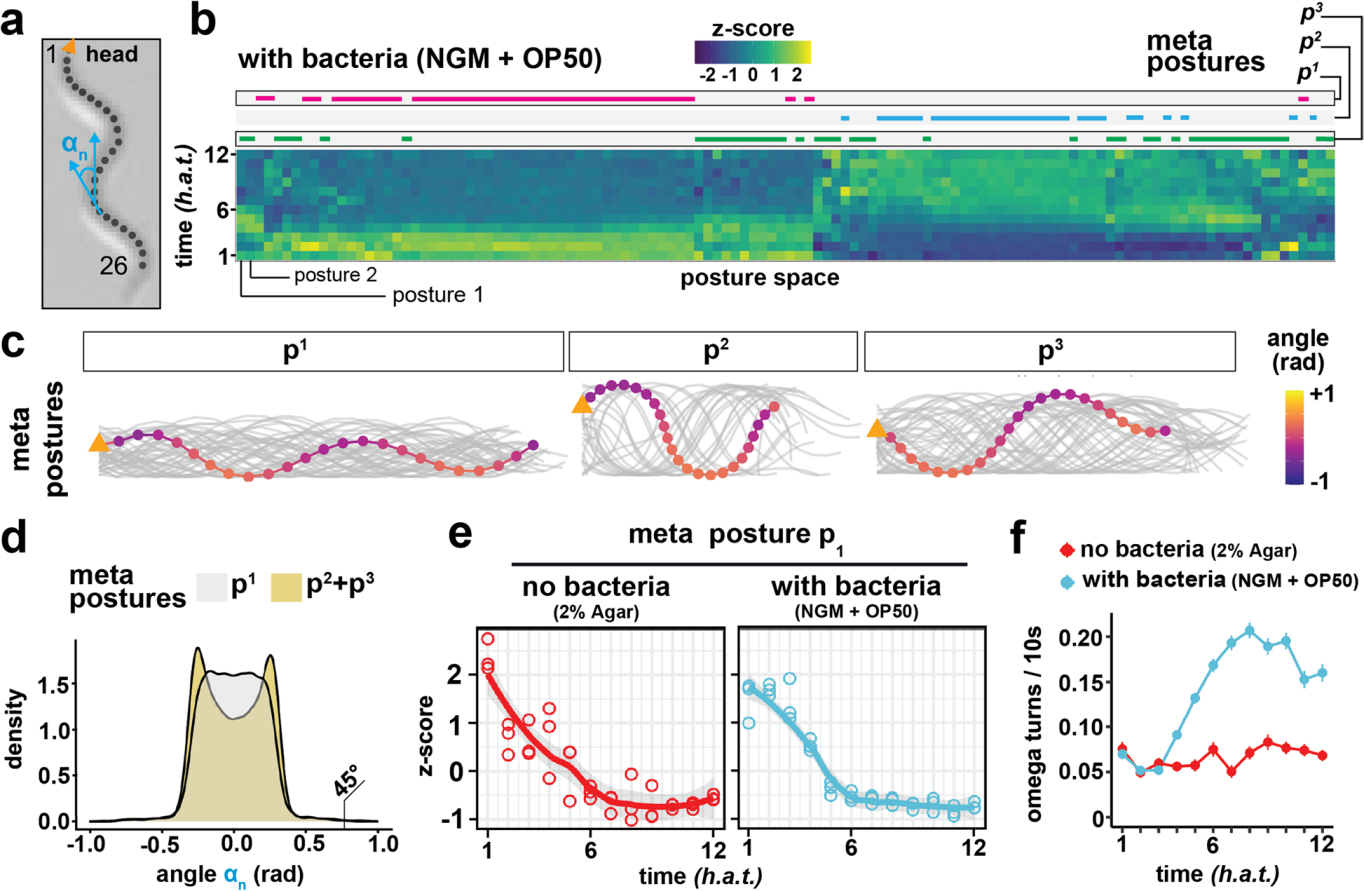
Postural adaptation during dauer exit. **a** Schematic of a worm skeleton. For each skeleton, we calculate a vector of 24 intersegment angles that describe the worm’s posture. **b** Heatmap of the relative occurrences of 120 postures during dauer exit on a food source as quantified from *n* = 3 experiments. Color indicates whether a posture is over or underrepresented at that time point relative to all time points. The upper bars indicate three meta posture groups, each comprising several postures, and their allocation within the heatmap. Meta postures were obtained by a second k-means clustering with *k* = 3 of all postures from all experimental conditions. **c** The three meta postures that were calculated across all conditions. All postures of one group are depicted in gray and one example posture is shown in color. **d** Distribution of all intersegment angles, calculated as shown in **a**. The dauer-specific meta posture p^1^ is compared to the two other meta postures p^2^ and p^3^. p^1^ contains 46 postures, p^2^ and p^3^ contain 28 and 46 postures, respectively. **e** Relative occurrence of the meta posture p^1^ during dauer exit, normalized to all time points of the depicted experimental condition. Each dot represents one experiment of *n* = 3 experiments. **f** Turn frequency during dauer exit, quantified with a custom random forest classifier (see Materials and Methods). Depicted is the mean, error bars are SEM

*C. elegans* posture, i.e., the worm’s shape, has been previously established as a robust measure for inferring behavioral motifs based on environmental stimuli or genetic differences [30–35]. Specifically, we hypothesized that the dauer-specific stiff posture characterized by shallower bends would disappear during dauer exit. Moreover, we asked the question whether the dauer-specific dispersal phenotype would be reflected at the posture level. As previously described for adult worms [35], segmented worm shapes extracted from moving worms across all time points have low complexity and can be explained by a combination of eigenvectors obtained from the covariance matrix of intersegment angles (Fig. 3a), so-called Eigenworms (Additional file 1: Supplemental Figure S3). We created a dauer-specific posture library (i.e., a postural syntax) for each condition [34] and plotted relative posture abundances across all time points (Fig. 3b, Methods). Posture abundances during dauer exit were consistent across experimental repeats (Additional file 1: Supplemental Figure S4). Interestingly, we detected a shift in postural syntax at about 6 *h.a.t* when dauers were shifted to a bacterial food source (Fig. 3b). We next aimed at further characterizing postural syntax dynamics across conditions and performed a second round of clustering on the previously defined posture libraries to identify the 3 most diverging posture groups (i.e., meta postures, p^1^, p^2^, p^3^) and compare their relative abundance across conditions (Additional file 1: Supplemental Figure S5; Supplemental Figure S6). While skeletons of the first meta posture p^1^ resemble the dauer-specific constricted posture, p^2^ and p^3^ contain more bent postures (Fig. 3c). Consistently, intersegment angle distributions differ significantly between meta postures (Fig. 3d). Next, we quantified the relative abundance of the dauer-specific p^1^ postures across time points and noticed a decrease in relative abundance within the first 6 *h.a.t.* in all tested conditions except for d*af-2* mutants that exhibited an impaired transition (Fig. 3e and Additional file 1: Supplemental Figure S6). It is important to note that due to our skeletonization implementation (Methods), our posture libraries did not contain overlapping postures of turning worms. Hence, the observed decrease in dauer specific postures could be the result of an increase in turn frequency as observed in starved worms [36]. To test this possibility, we trained a random forest classification model to identify turn postures (Methods; Additional file 1: Supplemental Figure S7) and quantified the fraction of turning worms during dauer exit. While the fraction of turns increases in the +OP50 NGM condition ("with bacteria"), turn frequency remains low on 2% Agar plates ("no bacteria") (Fig. 3f). We conclude that the measured posture switch detected under conditions with and without a bacterial food source cannot be solely explained by an increase in turn frequency. Importantly, *“with bacteria”* and *“no bacteria”* assay plates differed not only by bacteria concentration but also salt and additive concentrations that might also impact dauer behavior (see Methods). However, a posture switch was detected in all tested conditions early during dauer exit, highlighting the robustness of the detected food-independent behavioral switch. Taken together, we detect a new example of behavioral adaptation that consists in switching from a dauer-specific stiff and thin posture to a more bent, canonical *C. elegans* posture. This change in postural syntax happens within the first 6 *h.a.t.*, but is independent of the presence of a food source, does not explain the dauer-specific dispersal behavior and might be driven by other environmental factors (e.g., pheromone concentration).

### Genes driving behavioral changes during dauer exit

**Fig. 4:**
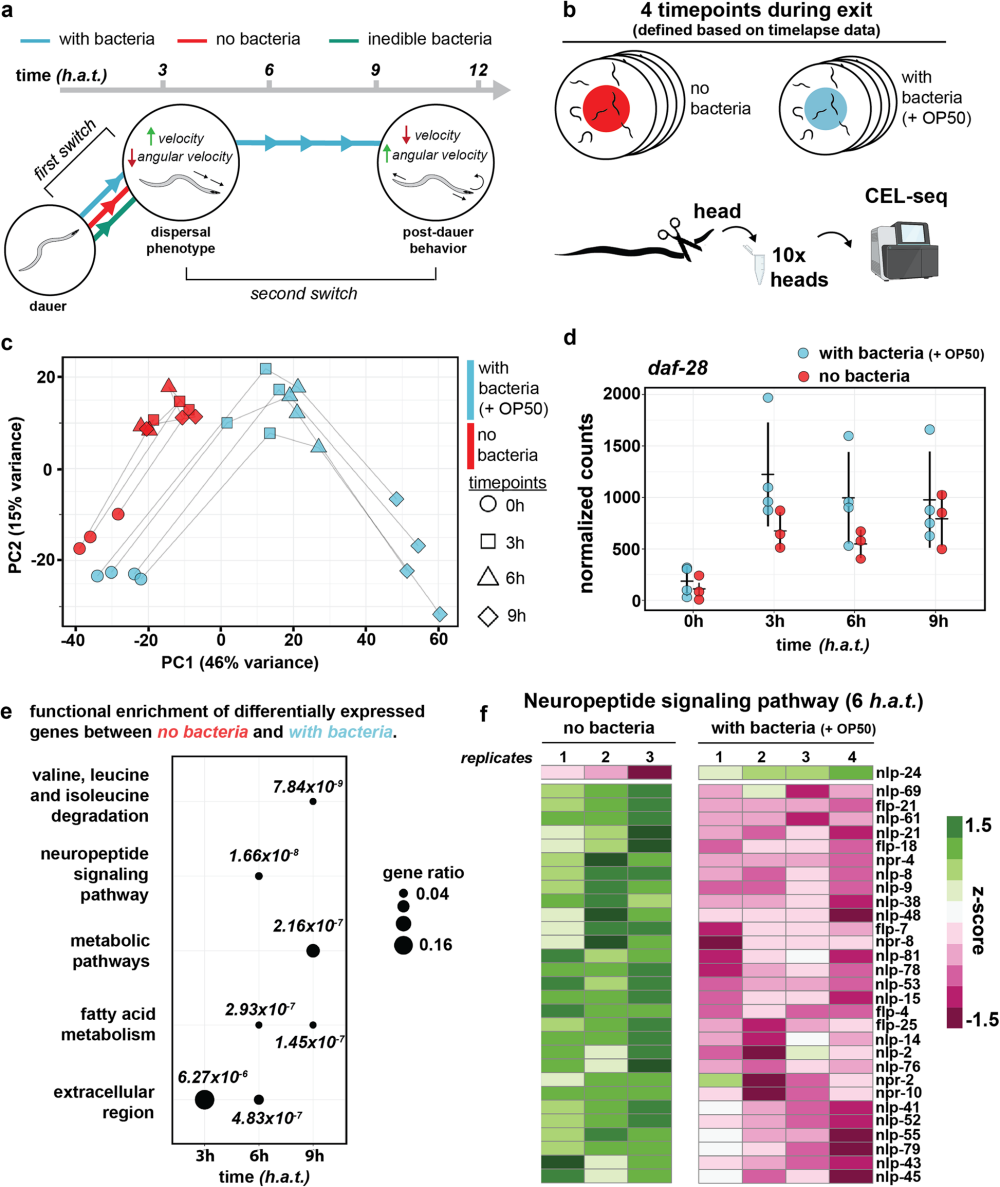
Transcriptome profiling reveals dynamic neuropeptide signaling during dauer exit. **a** Schematic summarizing the two main behavioral switches identified by behavioral imaging. **b** Schematic of the head-specific low-input mRNA sequencing method. For each time point and each condition, 10 heads were sequenced. **c** PCA of all samples, using the log transformed and normalized counts of the 500 most variable genes as input. **d** Normalized counts of *daf-28* across both conditions and all time points. Depicted is the mean ± standard deviation. **e** Dotplot depicting the result of the GO-term analysis. For each time point, differentially expressed genes between the two conditions were determined, and the result was used for GO-term analysis. Size of the dot scales with gene ratio. Gene ratio refers to the proportion of genes in the input list that are annotated to that GO-term. Shown are the top 3 GO-terms for each time point, and *p*-values are indicated next to each dot. **f** Heatmap of genes contained within the “neuropeptide signaling pathway” GO-term, comparing both conditions at 6 *h.a.t.* One column shows one experimental repeat

Next, we asked the question whether the two main behavioral switches quantified by imaging (Fig. 4a) would also be reflected at the level of gene expression, potentially through the expression of specific neuropeptides, receptors, and ion channels in neurons, previously described to be sufficient to cause drastic behavioral changes in *C. elegans* [37–39] and dauers specifically [9, 40]. For instance, insulin-like peptides (ILPs), which are expressed in the head of the worm [41], have been previously shown to be implicated in regulating dauer arrest [41–43]. We therefore performed a low-input RNA sequencing method [44] on *C. elegans* heads specifically at 4 different time points during dauer exit, exposing recovering dauers to an environment with or without a food source, thereby mirroring the conditions used for behavioral analysis (Fig. 4b, Materials and Methods). Next, we asked the question whether the behavioral switches were recapitulated at the level of the transcriptome by plotting gene expression in PCA space across all conditions and time points. Strikingly, for the condition lacking a food source, head-specific transcriptomes of 3 h, 6 h, and 9 h time points were similar as indicated by PCA clustering (Fig. 4c). Analogous to the behavioral data, if bacteria were provided, the time points 6 h and 9 h separated as well (Fig. 4d), indicating a two-step process driving the exit decision. We next wanted to characterize both behavioral adaptation switches in greater detail by identifying differentially expressed genes between the two conditions at different time points. Here, we focus on transcripts that are relevant for the behavioral adaptation process, but we offer an interactive online resource [45] for exploring the whole dataset. To characterize differential expression during the first food-independent behavioral switch, we computed a list of genes that were differentially expressed in both conditions when comparing the 0 h and 3 h time points. This revealed a list of genes, which contained all members of the arcasoid beta-oxidation pathway [46], all being downregulated in the first 3 *h.a.t.* (Additional file 1: Supplemental Figure S8). Additionally, we noticed significant upregulation of the insulin-like peptide *daf-28*, described to be transcriptionally repressed by increased pheromone concentration [43, 47] and required for dauer exit [48]. Taken together, we note that early in dauer exit, potentially driven by changes in pheromone concentration (e.g., through downregulated beta-oxidation pathway activity), *daf-28* expression goes up. This change in *daf-28* expression is independent of the presence of a food source and therefore indicative of an early, intermediate behavioral state that takes place while the exit process is still ongoing.

Next, we sought to investigate the second food-dependent behavioral switch that depends on a bacterial food source, which was already apparent at the transcriptome (Fig. 4c). To identify biological pathways that are differentially regulated between with and without bacteria conditions, we performed functional enrichment analysis using differentially expressed genes as input (Fig. 4e, Methods). At 6 *h.a.t*, coinciding with the second behavioral switch to post-dauer behavior, the most significantly differentially expressed gene ontology (GO) term was “neuropeptide signaling pathway” (p-value: 1.66x10^-8^) (Fig. 3f). The differentially expressed genes within this GO term revealed numerous neuropeptides, comprising both FMRFamide peptides (FLPs) and neuropeptide-like proteins (NLPs), previously described to modulate behavior in *C. elegans* [49] as well as some of the corresponding receptors (Additional file 1: Supplemental Figure S8). We found that almost all (23/24) neuropeptides were downregulated during dauer exit. Strikingly, one neuropeptide, *nlp-24*, was upregulated (Fig. 4f). Besides NLPs and FLPs, we also note differential regulation of ILPs, the third class of neuropeptides in *C. elegans*, at 6 *h.a.t* when comparing environmental conditions (Additional file 1: Supplemental Figure S8). Consistent with insulin signaling driving successful dauer exit, *ins-17*, a DAF-2 antagonist [50], is downregulated, while the counteracting *ins-4*, a DAF-2 agonist [47], is upregulated. Changes in neuropeptide gene expression are followed by other downstream programs initiated during dauer exit like for instance molting as revealed by GO-term analysis between 6 h and 9 h time points (Additional file 1: Supplemental Figure S9). In summary, we find that dauers that have been placed in an environment containing a food source will exhibit a behavioral change after 6 *h.a.t.*, which coincides with a drastic change in neuropeptide gene expression.

## Discussion

In this work, we present the WormObserver as a new, easy-to-use, and cost-effective open hardware solution for the analysis of *C. elegans* behavior. We envision our method to be useful for many labs independent of computational expertise to perform long-term acquisitions and analysis of *C. elegans* behavior. A description of the workflow, including detailed instructions, can be accessed under [22]. The modular nature of our system allows for the integration of more advanced opto-electronics or software components (e.g., optogenetics [51] or other tracking software [52, 53]). Moreover, given its cost (< 150$) and straightforward assembly, the platform can be considered for larger screens involving several microscopes running in parallel. We also note that in the future, one might consider running parts of the analysis directly at the microscope to reduce data footprint and analysis time, for instance by only saving worm centroid locations. As cost-effective GPU-equipped minicomputers similar to the RaspberryPi are emerging (e.g., NVIDIA Jetson), application of custom deep learning models to perform these tasks directly at the microscope becomes feasible.

Using the WormObserver, our behavioral analysis of dauer exit revealed two behavioral adaptations that have not been described previously (Additional file 1: Supplemental Figure S10):

1. When placed into a new environment, dauer larvae will exit locomotion quiescence and move into a highly mobile, dispersal state. In parallel, dauers will abandon the dauer-specific narrow posture. This first behavioral adaptation happens within 6 *h.a.t* and is independent of the presence of food. In the wild, dauers can overcome long distances and disperse to more favorable environments by phoresy, the ability to be carried on by another animal [26, 27]. However, an intermediate, highly mobile behavioral state occurring early in the exit decision process would enable fast dispersal to more favorable environments across smaller distances. This hypothesis is supported by the fact that *C. elegans*’ “boom-and-bust” lifecycle in the wild crucially depends on active migration during the dauer stage [54]. Interestingly, dauers exhibit distinct alterations in muscle structure, notably increased muscle arm extension, which may facilitate dispersion [55]. Importantly, in this work, we are measuring behavioral phenotypes within a population of dauer larvae. Hence, we are potentially missing minimal variability in terms of exit behavior at the level of individual specimens. It will be very interesting to further assess this variability for characterizing underlying robustness in exit-decision making (e.g., with single worm imaging in multi well set-ups [56, 57]). In summary, we hypothesize that the ability to switch to a highly mobile dispersal phenotype is likely to have evolved as an additional behavioral strategy to increase the dauer larva’s ability to efficiently disperse to more favorable environments. Strikingly, co-occurring with this first behavioral adaptation, we notice the upregulation of the only insulin-like peptide that is required for dauer exit, DAF-28. Moreover, *daf-28* is expressed in ASJ neurons [43], the critical sensory neuron for dauer exit [58]. Interestingly, during *C. elegans* L1 arrest exit, food perception triggers *daf-28* upregulation [59]. Moreover, similar to what we observe during dauer exit, *daf-28* upregulation primes L1 larvae for food update but is not sufficient for successful exit. Here, we show that during dauer exit, *daf-28* expression increases independently of the presence of a bacterial food source (Fig. 4d). Taken together, it will be interesting to further explore *daf-28*’s role as a transcriptional output of environmental cues and its contribution to exit decisions in both L1 and dauer larvae. More generally, using the WormObserver as a cost-effective tool for long-term behavioral experiments, it will be interesting to obtain behavioral data for all mutants of the dauer pathway (e.g., daf-7, daf-9, and daf-12).
2. If the new environment contains a food source, at 6 *h.a.t.*, dauers, which have resumed food ingestion at this point, start to ingest bacteria and eventually leave the transient dispersal-like state to adapt post-dauer behavior. Strikingly, we find that the two transitions observed at the behavioral level are both reflected at the transcriptome level as well. We also discovered that neuropeptide transcription is regulated in response to food during dauer exit, potentially initiating behavioral adaptation. Neuropeptides are well-conserved, short amino acid sequences that can directly act as neurotransmitters but also have neuromodulator capacity [60]. In *C. elegans*, the dynamic expression of neuropeptides has been described to control several fundamental behavioral programs, e.g., mating [61], feeding [38], sleep [62], and others [49]. More specifically, in the context of dauer larvae, previous work has identified increased neuropeptide signaling as an important neuromodulatory event for promoting the dauer entry decision [63, 64]. Hence, adjusting the neural transcriptome at the level of diffusible signaling-molecules, i.e., neuropeptides, increases the larva’s behavioral repertoire. However, we also find that some neuropeptides are specifically upregulated during dauer exit on a food source (e.g., *nlp-24*, *ins-4*), highlighting the complexity of neuropeptide signaling. Given its previously reported role in mediating pharyngeal pumping in starved worms [65], upregulation of *nlp-24* could regulate onset of pharyngeal pumping during dauer exit but further experiments will be needed to confirm this hypothesis. Previously, NAD+, which is contained within the bacterial food source, has been shown to promote pharyngeal pumping in dauer-exiting larvae [66]. Here, we show that only ingestible bacteria will later in the process promote another behavioral switch affecting locomotion. It will be interesting to further characterize which components of the ingested bacteria are required for this process, how other environmental factors (e.g., salts and organic material) influence dauer exit and how these components are sensed and processed. Furthermore, in this study, we do not perform gene expression analysis at the level of individual neurons although gene expression changes in single neurons have been described to initiate changes in *C. elegans*’ developmental trajectory. For instance, the DAF-2 agonist *ins-6*, has been shown to promote dauer exit by switching between the two sensory neurons ASI and ASJ [42]. Thus, we note that, in the future, resolving transcriptional dynamics of neuropeptides at the level of individual neurons (e.g., through single-cell RNA sequencing [67, 68]) will further improve our understanding of the underlying circuit adaptations.

## Conclusion

In summary, while comprehensive studies of the combined effect of neuropeptide gene expression changes are missing, our study sheds light on the role of neuropeptides in establishing a developmental decision in response to environmental stimuli. By correlating differential expression with long-term behavior data obtained in the same conditions, we associate gene expression changes to behavioral switches during dauer exit, making a first step towards answering the question how nervous system plasticity can be achieved by dynamic expression of neuromodulators. Thus, our work also highlights how the combination of long-term behavioral imaging and gene expression analysis can improve our understanding of developmental and neuronal plasticity.

## Methods

### *C. elegans* strains and handling

Worms were maintained at 15 °C on nematode growth medium (NGM) with *Escherichia Coli* OP50 as food source as previously described. Dauer formation was induced by transferring a mixed population of ca. 50–100 worms to 6cm NGM plates seeded with *E. coli* OP50. Plates were then incubated for at least 7–10 days at 25 °C. Next, worms were washed off with M9, washed twice to eliminate remaining bacteria, and 1% SDS solution was added. The mixture was incubated for 30 min at room temperature [4]. After an additional washing step with M9 to eliminate SDS, worms were transferred to an unseeded NGM plate. After 30 min, once the drop of M9 dried out, dauers, which can survive SDS treatment (unlike all other *C. elegans stages*), crawled out of the mix of dead worms and were collected. All experiments were carried out with the N2 Bristol strain (obtained from CGC) except for experiments involving a *daf-2* insulin mutant. Animals carrying the *daf-2 (e1370)* mutation were incubated at 25 °C for at least 7 days to obtain pure dauers and subjected to the same SDS-selection procedure prior to imaging.

### Assay plates

We used commercially available freeze-dried *E.coli* OP50 (*+OP50*, available from LabTIE) or HB101 (*+HB101*, available from CGC) bacteria for conditions involving a bacterial food source. Dead OP50 bacteria were prepared according to the manufacturer’s instructions (final concentration: 3.6mg/ml). HB101 were cultured overnight at 37 °C. Both cultures were diluted 1:10, added to 3cm NGM plates, UV irradiated to abolish bacterial growth and kept for storage at 4 °C. Inedible bacteria were prepared by incubating *E.coli* OP50-GFP (available from CGC) with Aztreonam (Cayman Chemical, CAY19784-1) as previously described [69]. The resulting elongated bacteria were visually inspected under a fluorescence microscope, seeded on NGM medium, UV irradiated and kept for storage at 4 °C. For the “no bacteria” condition, plates contained only 2% Agar.

### SDS-resistance assay

N2 dauers were obtained as described above and placed on assay plates with or without bacteria (see above). Every hour, ca. 50–100 worms were washed once in M9, added to 1% SDS, and incubated for 15 min at room temperature. Next, an additional wash in M9 was performed and worms were checked for viability on an unseeded NGM plate.

### WormObserver and imaging

For each imaging experiment, ca. 250 dauers were transferred in a small drop of M9 and added to 3 cm plates (for preparation see above). We used a Zeiss Discovery V8 stereoscope with illumination from below the plate and a Plan S 1x objective with 3.2x zoom. To filter out blue light from the illumination source, a red filter (Edmon Optics, *#53-699*) was placed in between the light source and the assay plate. The WormObserver hardware consists of two parts: a custom 3D-printed eyepiece sleeve with a fixed 8 Megapixel Raspberry Pi camera and an attached Raspberry Pi model 3 with a touchscreen display for defining acquisition parameters. The Raspberry Pi camera is an 8 Megapixel Raspberry Pi V2 camera module with a maximum resolution of 3280 × 2464 pixels. All videos analyzed in this paper were acquired at a resolution of 1024 × 1024 pixels. Given our magnification (that is defined by the stereoscope’s objective and the eyepiece), we obtain a pixel size of 6.25 μm/px. Field of view is 6400 μm × 6400 μm. We provide all details for fabricating the hardware components together with the required software and descriptions under https://github.com/Fritze/WormObserver/. For all experiments shown here, we recorded at least 100 consecutive videos of 8 min (> 13 hours). Worms are allowed to move in and out of the field of view and the same worm might be captured multiple times. Therefore, our measurements should be considered a population average rather than “single worm” measurements. All timelapse data was acquired at room temperature (20 °C). Experimental repeats were performed on different days.

### Image analysis

#### Imaging and tracking

The custom, open-source KNIME workflow that is designed for automated processing of the data can be assessed under https://github.com/Fritze/WormObserver/tree/master/KNIME. Users only need to define the location of the incoming videos as sent by the RaspberryPi, and all image processing steps are performed automatically with no user intervention needed. H264-compressed videos are automatically converted into images using ffmpeg and downsampled from 5 fps to 2 fps for tracking. This downsampling step is optional and can be omitted if larger frame rates are required. Briefly, worms are segmented after background subtraction, filtering, and size selection of segmented connected components. Tracks are identified using the TrackMate [21] node and worm skeletons extracted using the “skeletonize” ImageJ command in KNIME. In summary, each video of a given timelapse experiment is summarized in a result table containing an ID, the relative position, track statistics, turn prediction score, the segmented shape, and the skeleton for all detected and tracked worms.

A workstation (Intel 2.6 GHz CPU, 64 GB RAM) was used for processing all datasets. Processing 100 time points took about 17 h per dataset, which is only 4 h longer than the runtime of the timelapse, highlighting the value of parallelizing acquisition and analysis for long timelapse experiments.

#### Tracking data analysis

We wrote custom code in R to process result files as given out by the KNIME workflow. All analysis steps presented in this paper can be performed by calling R-scripts from the command line. All scripts can be accessed under https://github.com/Fritze/WormObserver/tree/master/code/tracking. For all analysis, we discard the first 30 min of the timelapse to avoid artifacts introduced by worm handling. Briefly, we calculate speed and angular velocity per track over 10 s bins. Only tracks without gaps and of minimum length of 20 frames (i.e., 10 s at 2 fps) were considered. We additionally exclude non-moving tracks to avoid segmentation artifacts. Dispersal behavior was defined as tracks having a minimum speed of 0.01 mm/s and angular velocity below 15 °/s.

#### Skeletonization

For every frame and segmented worm, a skeleton was extracted and saved from within the KNIME workflow.

All subsequent analysis steps were performed in R and relevant source code can be accessed under https://github.com/Fritze/WormObserver/tree/master/code/tracking/. Briefly, for each skeleton, we first estimate the worm’s head position based on the direction of movement. Next, only skeletons that can be unambiguously identified (i.e., no branches) and of a minimum length of 26 pixels are retained. Finally, for skeletons where the first step could not assign head position on the direction of movement, information from adjacent frames was used. If adjacent frames did not contain head position information, the skeleton was discarded. We manually confirmed this approach to be a good conservative estimate of the worm’s skeleton and head position. For calculating *Eigenworms*, we focused on moving worms and only included tracks with a minimum track duration of 5 s, speed > 0.01 mm/s, and angular velocity < 50°/s.

#### Posture analysis

For each skeleton, we then fit 26 equally spaced segments on the midline of the worm and calculate a vector of 24 intersegment angles, previously shown to be sufficient for describing *C. elegans* posture [32]. To cluster postures based on intersegment angles obtained after skeletonization, we used an approach adapted from [34]. For this analysis, we only considered tracks that were successfully tracked and skeletonized for at least 5 s without interruption. Since we do not follow individual worms and therefore image at lower magnification to capture a larger number of larvae within the field of view, we could not distinguish dorsal and ventral turns in our data. After kmeans clustering with *k* = 200, we next identified mirrored posture pairs and treated each pair as if it was the same posture for further analysis. This reduced the complexity of our posture library by ca. 50% depending on the analyzed condition. This first round of clustering was done individually for each condition but across experimental repeats. Similarly, the *z*-score depicted in Fig. 3b was calculated using the relative abundance of each posture (column in the heatmap) across all experimental repeats and time points (rows in the heatmap). For this calculation, all experimental repeats were combined and treated as if they were one experiment. To exclude batch effects, *Z*-score calculations of separated experimental repeats were performed as well (Additional file 1: Supplemental Figure S5). To compare relative abundances of certain posture types (i.e., meta postures) across conditions, we subjected all previously identified postures to a second round of k-means clustering (*k* = 3) and plotted the relative abundance of the three meta postures across conditions and experimental repeats. We note that the resulting posture libraries did not contain any self-occluded shapes (i.e., turns) as these did not pass our criteria for successful skeletonization. To be able to measure the abundance of turns in our datasets across time points and conditions, we used 17 image features extracted from segmented worm bitmasks and trained a random forest model for detecting turns. When training the random forest model, worm postures were annotated as “turn” when head or tail were touching the worm’s body or if both head and tail were clearly pointing in the same direction (U-shape). The model (incl. Bitmasks used for training) is available under https://github.com/Fritze/WormObserver/tree/master/KNIME and can be loaded into KNIME for inspection. The classification is integrated within the image processing workflow in KNIME and flags every frame of a track with either 0 or 1 if a turn was detected. Next, we bin all turn occurrences over a period of 10 s to avoid over counting still animals (i.e., if more than one frame is flagged as turn within the 10 s window, it will be counted only once).

### RNA sequencing

#### Library preparation

N2 dauer larvae were cultured and extracted as described above. For time point 0, dauers were directly collected from the unseeded NGM plate. Dauers assigned to “no bacteria” condition were always collected first and “with bacteria” condition second (10–20 min later). Assay conditions were the same as for the timelapse imaging experiments and both conditions (no bacteria and + OP50) were processed in parallel. Every hour, a fraction of dauers was washed off the assay plates and anesthetized with 1mM tetramisole. Under a dissecting microscope, the worm’s head was cut off just below the terminal bulb of the pharynx. For each time point, 10 heads were pooled into a pre-cooled Eppendorf tube on dry ice, containing 500 μl trizol and 1 μl GlycoBlue. Samples were frozen at – 80 °C for storage and later subjected to RNA sequencing according to the Tomo-Seq protocol [70] with minor modifications. Briefly, after thawing, samples were mixed thoroughly and incubated for 5 min at room temperature. Subsequently, 100 μl of chloroform were added to each sample, mixed well, and incubated for 5 min. Samples were then centrifuged at 12,000 g for 15 min at 4 °C. Next, the aqueous phase was carefully transferred to an Eppendorf LoBind containing 250 μl isopropanol and precipitated overnight at − 20°C. Samples were centrifuged for 10 min at 12,000 g and at 4 °C. Next, the supernatant was removed and the RNA pellet was washed with 75% freshly prepared ethanol. After washing, the pellet was dried and, once completely dry, resuspended in 2 μl molecular grade water. One microliter of the sample was transferred to a fresh low binding PCR tube containing barcoded oligo (dT) primer. Reverse transcription was performed with the Superscript II kit according to [70]. First strand synthesis was performed for 1 h at 42 °C. Second strand synthesis was performed for 1 h at 16 °C. Next, samples were pooled and purified with Agencourt AMPureXP beads. IVT was performed for 16 h at 37 °C. After fragmentation and purification, RNA was reverse transcribed with superscript II and amplified by PCR. Concentration and size distribution of the resulting cDNA were measured with Qubit and Tapestation. mRNA Libraries were sequenced on a NextSeq500 in paired end mode with 150 cycles.

#### Differential expression analysis

Raw sequencing basecalls were demultiplexed and converted to FASTQ format using custom python scripts. RNA-seq reads were mapped to the ce11/WBcel235 genome assembly using STAR_2.7.3a [71], annotated using Rsubread [72], and count matrices were generated using custom python scripts. PCA was done on normalized log transformed read counts of the 500 most variable genes across conditions and time points. Differential expression changes were determined with DESeq2 [73], with lfc shrinkage turned on. Differentially expressed genes were called with a significance cut-off of 0.05 (*p*-value adjusted) and a minimum log2 fold change of 1 or − 1. For GO term analysis, the gprofiler2 package [74] was used with a significance cut-off of *p*-value 0.01 and a custom background model that contained all genes that we detected with our method across all conditions and time points with at least 10 reads. The dataset can be interactively explored under https://www.bit.ly/dauer_exit.

## Supplementary Information

**Additional file 1.** Supplemental Figures S1-S9.

**Additional file 2.** Normalized transcript counts.

## Data Availability

All data generated or analyzed during this study are included in this published article, its supplementary information files, and publicly available repositories. Raw sequencing data and count tables have been deposited to GEO and can be accessed under GSE214208. RNA sequencing results data can be interactively explored at: https://www.bit.ly/dauer_exit. A full description of the WormObserver, including detailed instructions for all hardware and software components, can be found at https://github.com/Fritze/WormObserver. Raw data for all imaging experiments can be accessed under 10.25378/janelia.21463101.v1. All code used for analysis of tracking and sequencing data is available at https://github.com/Fritze/WormObserver/tree/master/code. The repository version used in this manuscript has been archived under *10.5281/zenodo.7108430*.

## Abbreviations

GUI: Graphical user interface
*h.a.t.*: Hours after transfer
NGM: Nematode growth medium
ILPs: Insulin-like peptides
PCA: Principal component analysis
GO: Gene ontology
FLPs: FMRFamide peptides
