# Supplementary material for: Long-term imaging reveals behavioral plasticity during C. elegans dauer exit
Source: BMC Biol. 2022 Dec 13;20:277. doi: 10.1186/s12915-022-01471-4 (PMC9749223; doi:10.1186/s12915-022-01471-4)
Supplement: Supplementary file 1 — Additional file 1. Supplemental Figures S1-S9. [file 12915_2022_1471_MOESM1_ESM.pdf]

Long-term imaging reveals behavioral plasticity during *C. elegans* dauer exit.

Friedrich Preusser[1,2,4], Anika Neuschulz[1,2], Jan Philipp Junker[1], Nikolaus Rajewsky[1], and Stephan Preibisch[3,1,4]

[1] Berlin Institute for Medical Systems Biology (BIMSB), Max Delbrück Center for Molecular Medicine in the Helmholtz Association (MDC), Berlin 10115, Germany

[2] Institute for Biology, Humboldt University of Berlin, 10099 Berlin, Germany

[3] Janelia Research Campus, Howard Hughes Medical Institute, Ashburn, VA 20147, USA

[4] corresponding authors

Supplemental figures

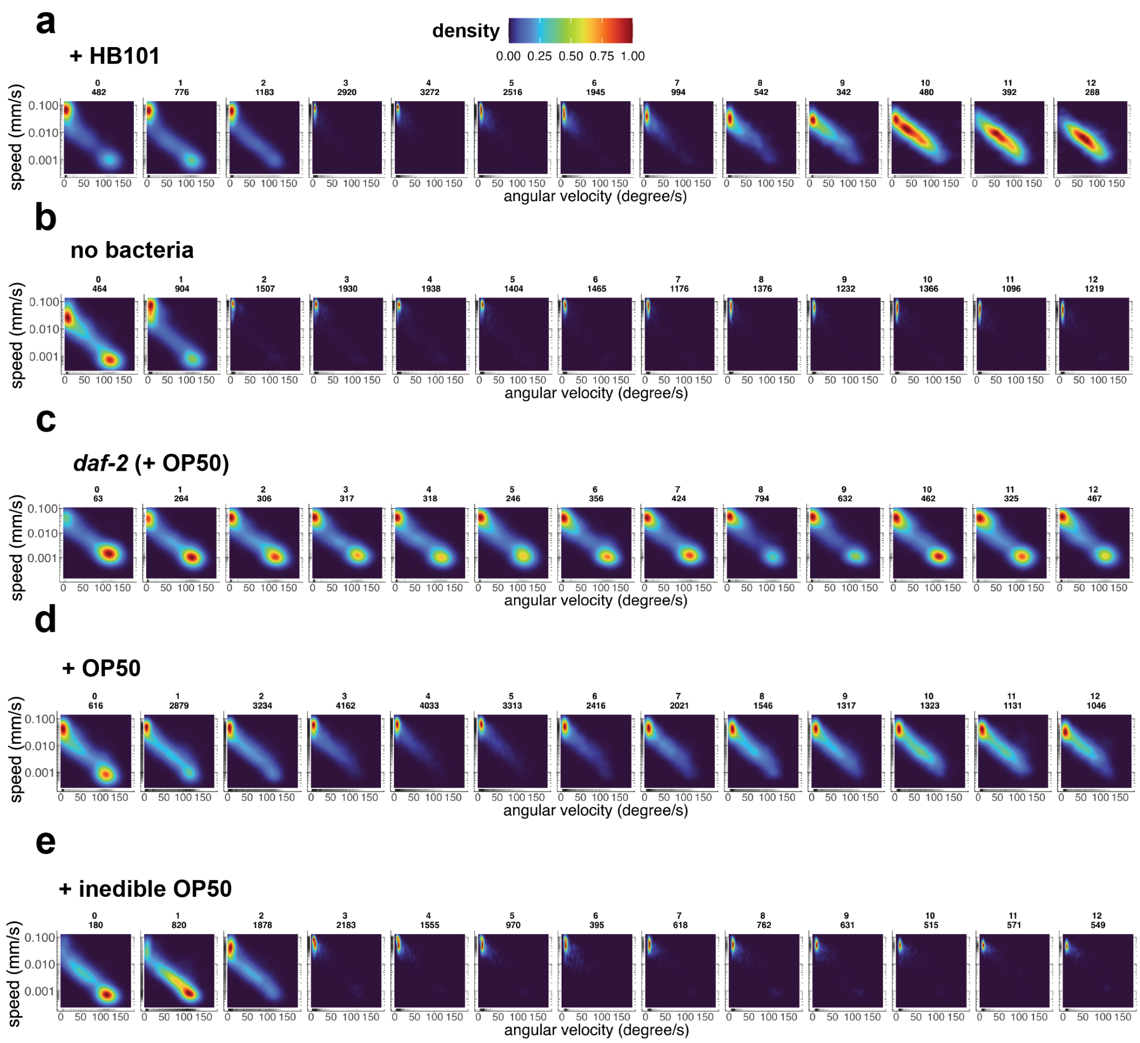

Supplemental Figure S1

(a-e) Two-dimensional probability distributions of speed (mm/s) and angular velocity (degrees/s) averaged over 10 s time windows across the first 12 hours of dauer exit in different environments. Indicated above the plot is the time after transfer and the number of tracked paths within that time point. OP50, no bacteria and inedible OP50 are data from n=3 experiments, all other n=2.

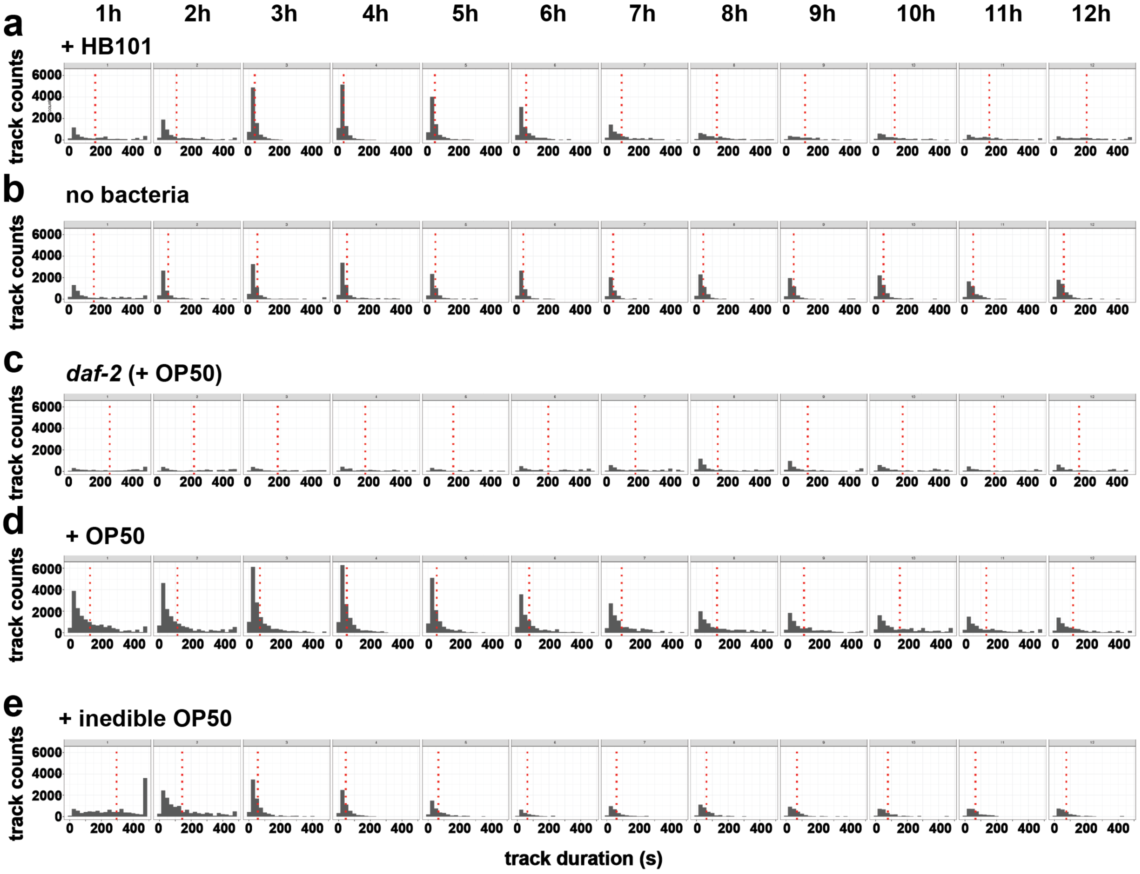

Supplemental Figure S2

(a-e) Histograms of track duration (s) for all tracks in each time point and condition. Mean track duration is indicated as dotted red line.

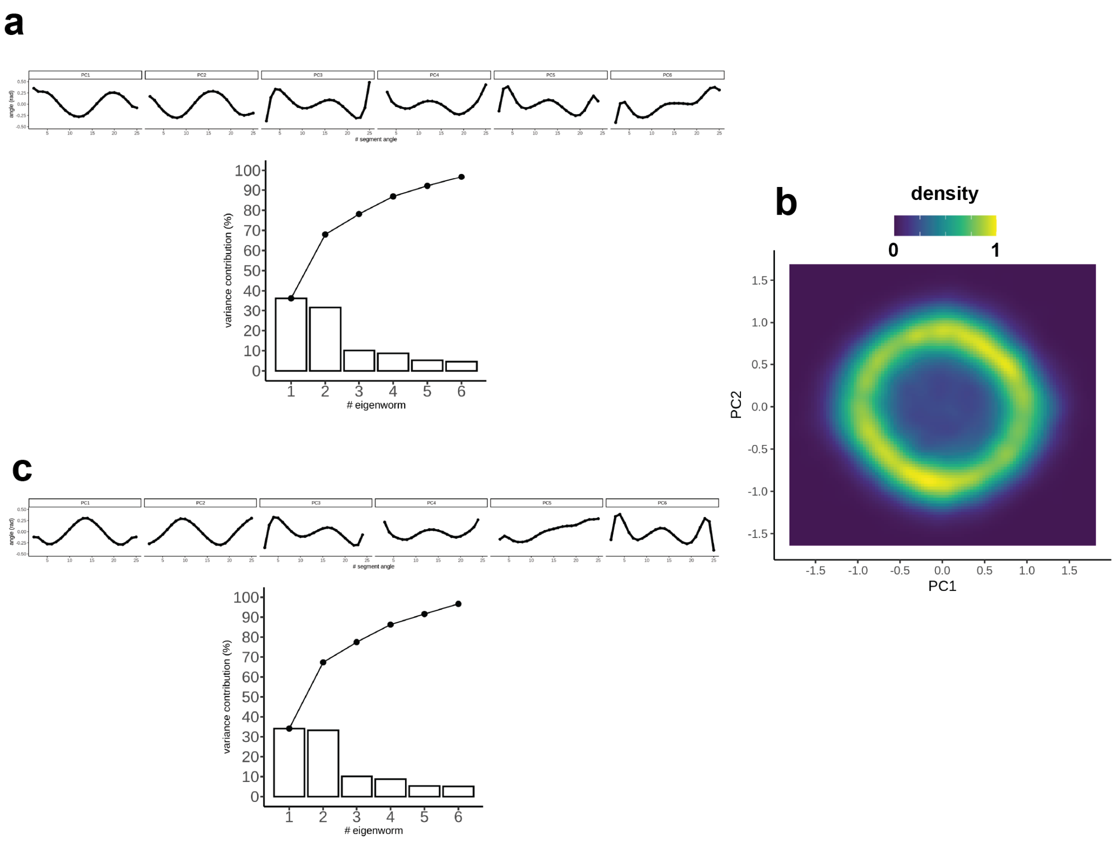

Supplemental Figure S3

(a) Top 6 *Eigenworms* for moving worms in the +OP50 dataset across all time points (n=3). Bar plot shows the variance explained by each *Eigenworm*. Traces show cumulative values.
(b) Probability density for the first two principal components.
(c) Top 6 Eigenworms of the same worms as shown in (a) but with randomized head and tail assignment.

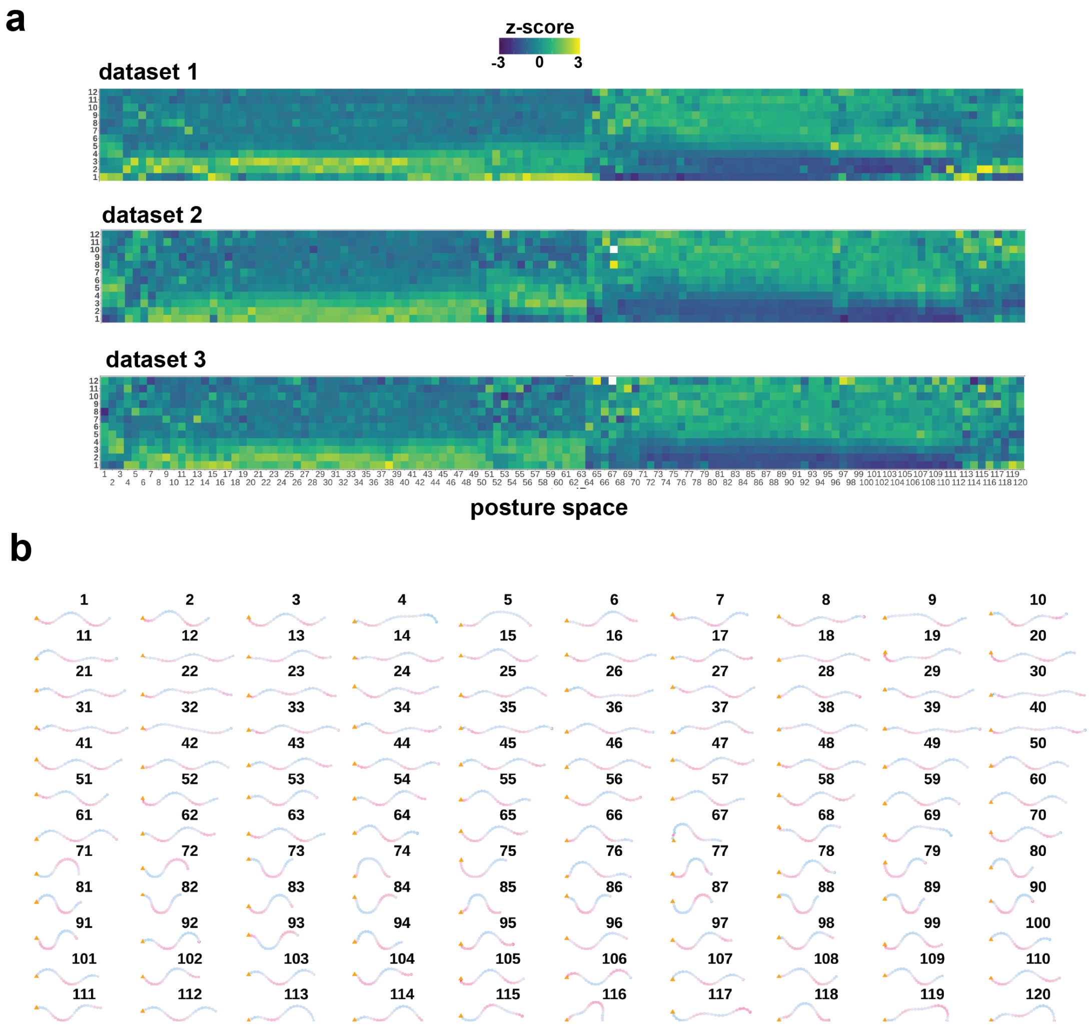

Supplemental Figure S4

(a) Heatmaps of the relative occurrences of 120 postures during dauer exit on a food source (+ OP50), plotted individually but within the same posture space for 3 experimental repeats. Color indicates whether a posture is over or underrepresented at that time point relative to all time points. The postures that are corresponding to columns in the heatmaps are plotted in **(b)**.

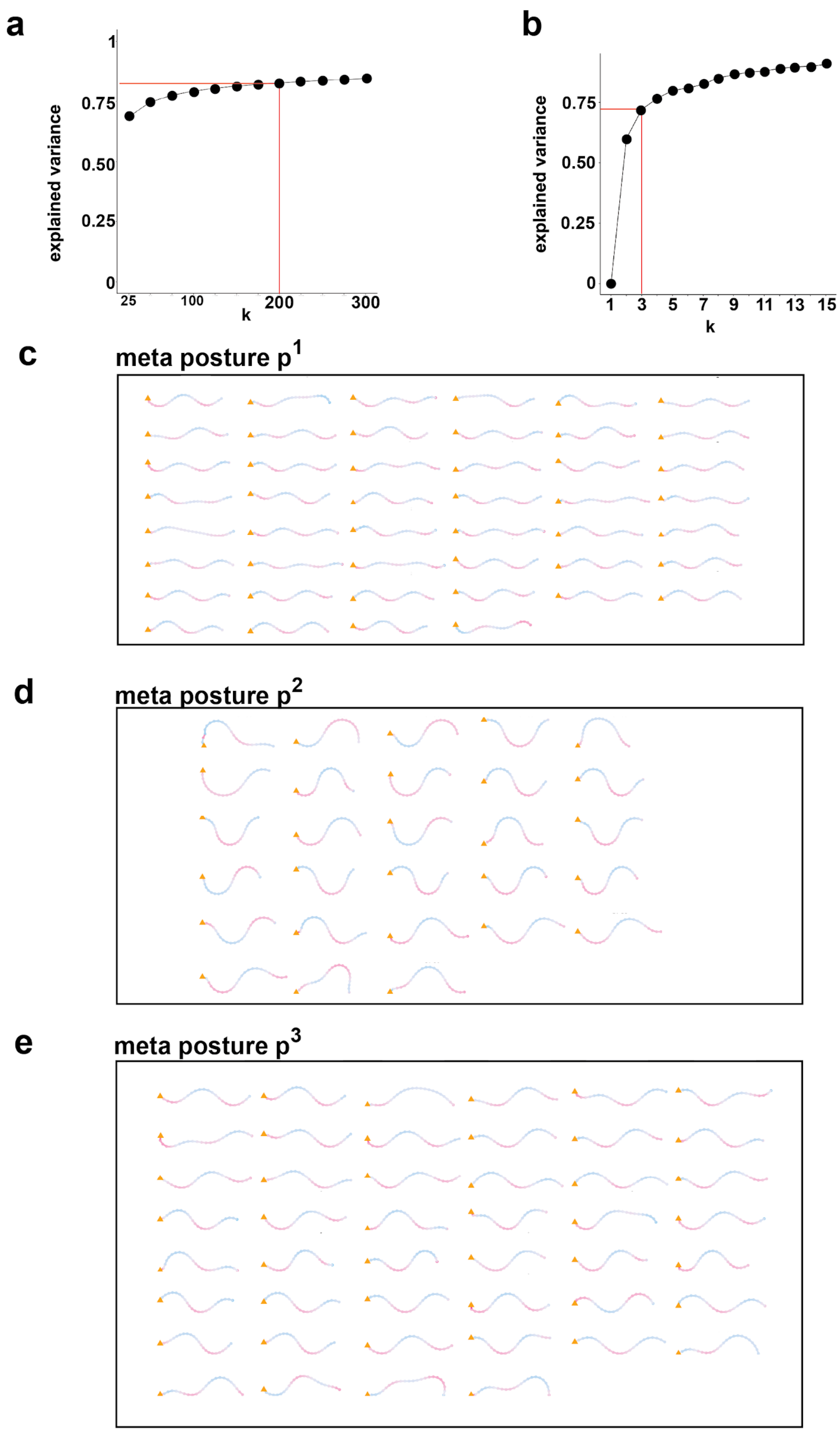

Supplemental Figure S5

(a) Elbow plot for posture identification through k-means clustering of angle vectors of all skeletons, showing the explained variance with increasing k. **(b)** Same as (a), but for meta posture clustering, using the previously determined postures as input. Red line indicates the used k in our analysis. **(c-e)** Postures contained within the three meta posture groups.

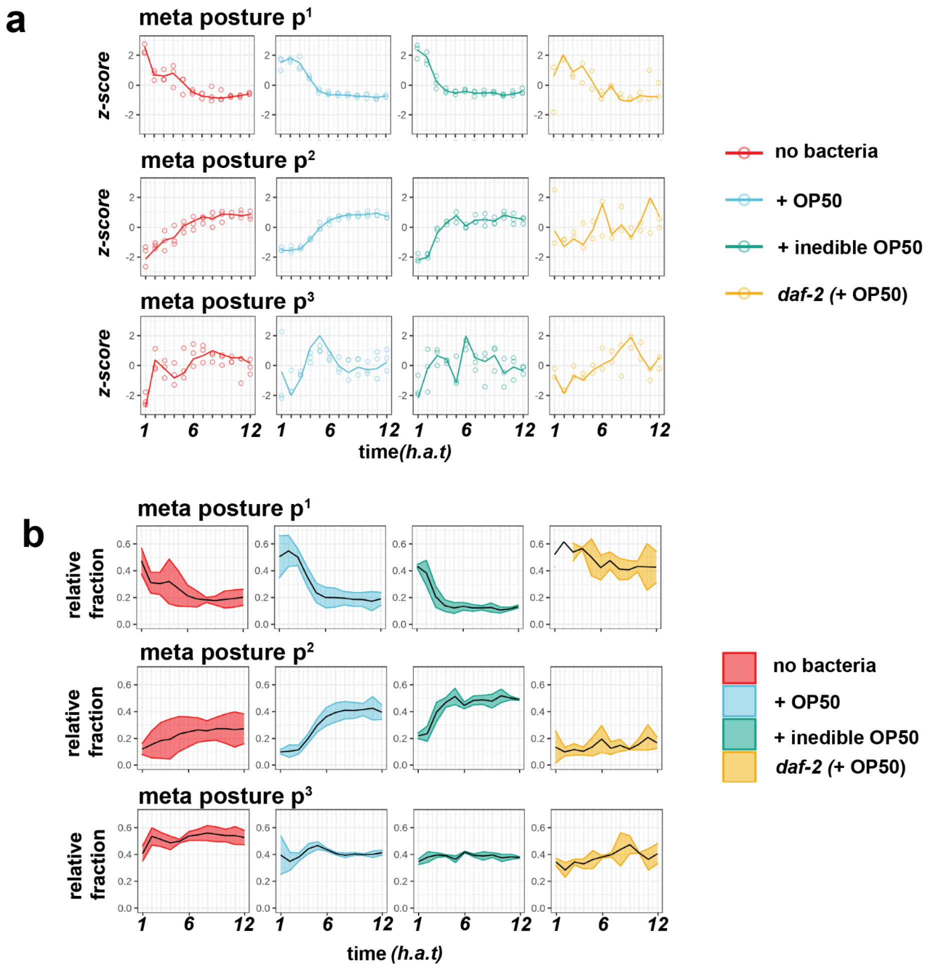

Supplemental Figure S6

(a) Relative occurrence of the three meta postures during the first 12 hours of dauer exit, normalized to all time points within one experimental repeat. Each dot represents one experiment of n=3 experiments (except *daf-2,* n=2). **(b)** Same data as in (a), but plotting the relative abundance of each metaposture across time and all experimental repeats of one condition.

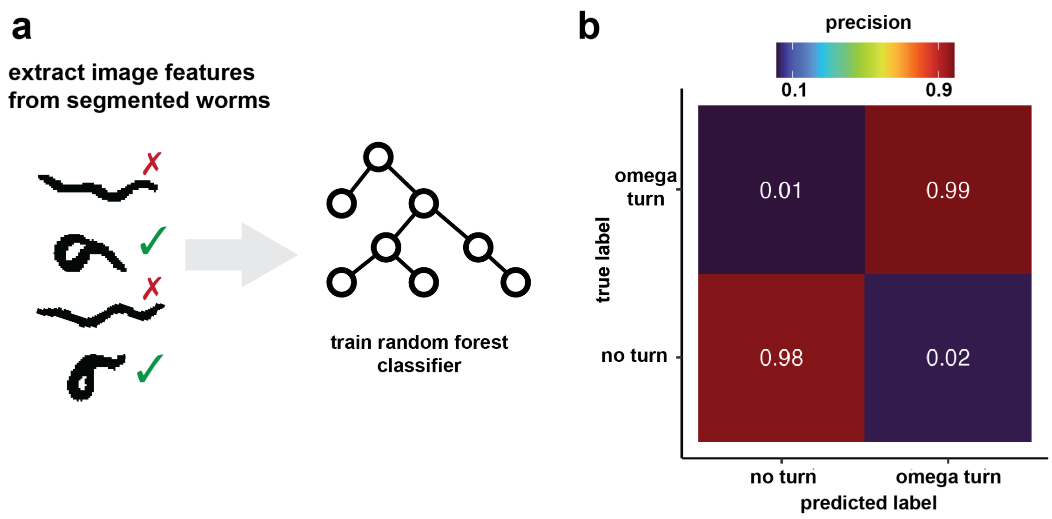

Supplemental Figure S7

(a) Schematic of the training process for the random forest classifier. 17 image features are extracted from labeled worm bitmasks and used for training the random forest classifier in KNIME. For new data, prediction will occur for each segmented worm and frame. **(b)** Confusion matrix showing the classifier's performance on a unseen test data set (93x "turn", 89x "no turn"). Indicated is the precision score.

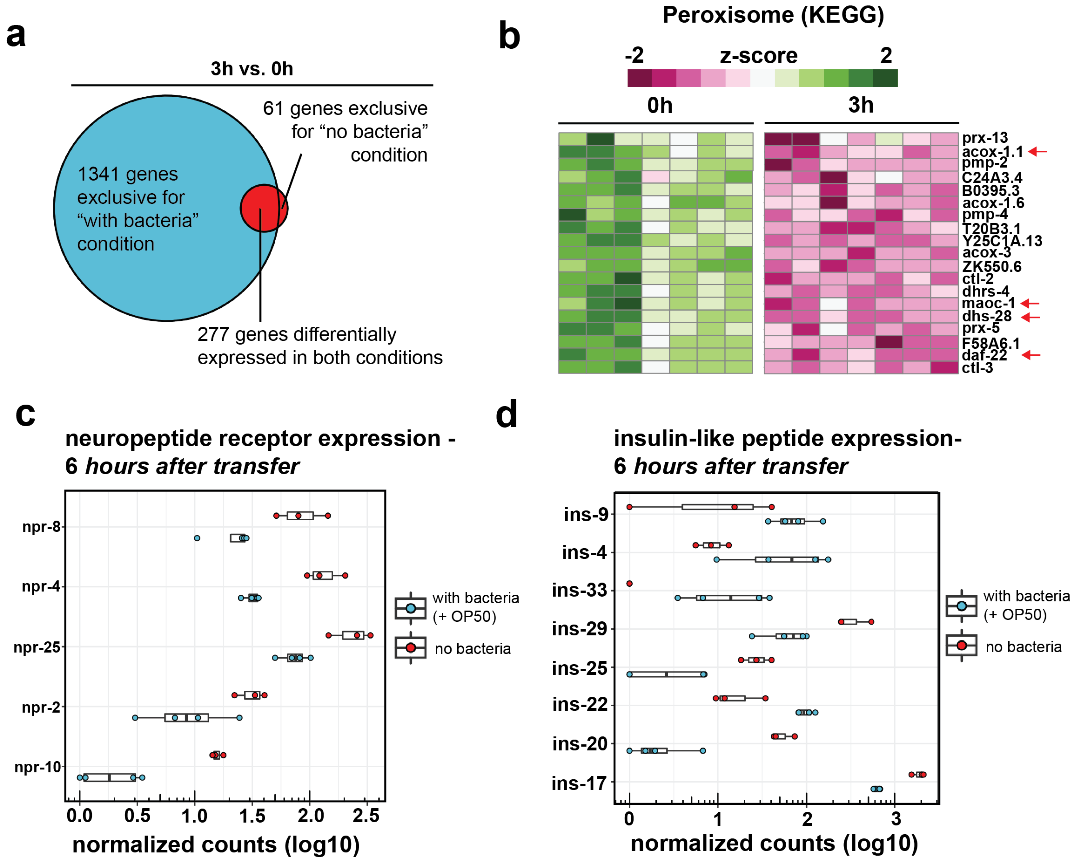

Supplemental Figure S8

(a) Venn-diagram showing the overlap between differentially regulated genes when comparing 0h and 3h time points either in a condition with (blue) and without (red) a bacterial food source. **(b)** Genes within the KEGG peroxisome GO-term, the third most significant GO-term when using the list of 277 genes that are differentially expressed in both conditions as input. Highlighted are the members of the peroxisome beta-oxidation pathway. **(c)** Normalized counts of differentially expressed neuropeptide receptors when comparing both conditions at 6 *h.a.t.* **(d)** Normalized counts of differentially expressed insulin peptides when comparing both conditions at 6 *h.a.t.* All shown genes were significantly different when comparing the two conditions (Wald test, p-value adjusted <0.05).

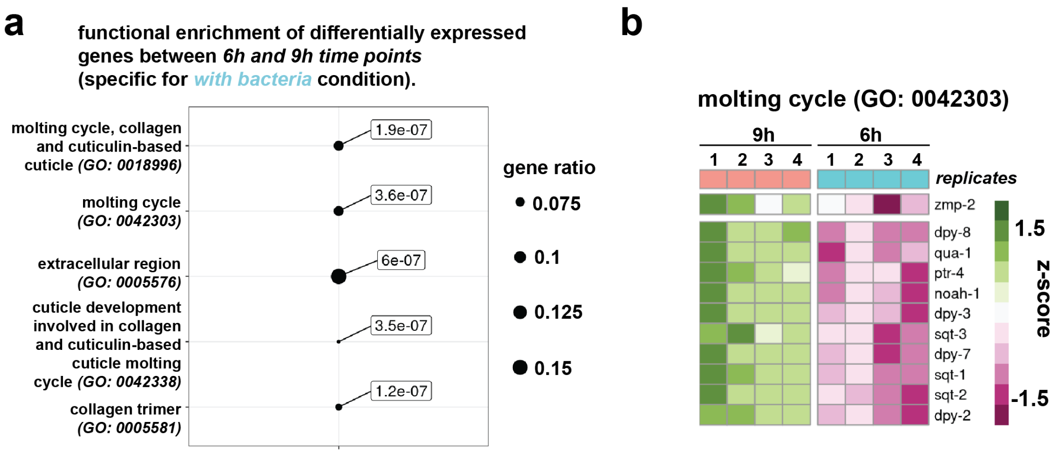

Supplemental Figure S9

(a) Dotplot depicting the result of the GO-term analysis for differentially expressed genes between 6h and 9h time points. Only genes that were specifically enriched in the "with bacteria" condition were used as input for GO-term analysis to reveal gene expression changes initiated by food ingestion. Size of the dot scales with gene ratio. Gene ratio refers to the proportion of genes in the input list that are annotated to that GO-term. P-values are indicated next to each dot. **(b)** Heatmap of genes contained within the "molting cycle" GO-term. One column shows one experimental repeat.
